# Glycemic deviation index: a novel method of integrating glycemic numerical value and variability

**DOI:** 10.1186/s12902-021-00691-z

**Published:** 2021-03-19

**Authors:** Yizhou Zou, Wanli Wang, Dongmei Zheng, Xu Hou

**Affiliations:** 1 Department of Endocrinology, Shandong Provincial Hospital, Cheeloo College of Medicine, Shandong University, 324 Jing 5 Road, Jinan, 250021 China; 2 Department of Endocrinology, Shandong Provincial Hospital Affiliated to Shandong First Medical University, Jinan, China; 3 Shandong Clinical Medical Center of Endocrinology and Metabolism, Jinan, China; 4 Institute of Endocrinology and Metabolism, Shandong Academy of Clinical Medicine, Jinan, China; 5 School of Mechatronical Engineering, Beijing Institute of Technology, Beijing, China

**Keywords:** Glycemic variability, Continuous glucose monitoring, Diabetes

## Abstract

**Background:**

There are many continuous blood glucose monitoring (CGM) data-based indicators, and most of these focus on a single characteristic of abnormal blood glucose. An ideal index that integrates and evaluates multiple characteristics of blood glucose has not yet been established.

**Methods:**

In this study, we proposed the glycemic deviation index (GDI) as a novel integrating characteristic, which mainly incorporates the assessment of the glycemic numerical value and variability. To verify its effectiveness, GDI was applied to the simulated 24 h glycemic profiles and the CGM data of type 2 diabetes (T2D) patients (*n* = 30).

**Results:**

Evaluation of the GDI of the 24 h simulated glycemic profiles showed that the occurrence of hypoglycemia was numerically the same as hyperglycemia in increasing GDI. Meanwhile, glycemic variability was added as an independent factor. One-way ANOVA results showed that the application of GDI showed statistically significant differences in clinical glycemic parameters, average glycemic parameters, and glycemic variability parameters among the T2D groups with different glycemic levels.

**Conclusions:**

In conclusion, GDI integrates the characteristics of the numerical value and the variability in blood glucose levels and may be beneficial for the glycemic management of diabetic patients undergoing CGM treatment.

## Background

Diabetic blood glucose disorders are mainly caused by abnormalities in the average glycemic level and variability, and the latter has been shown to independently affect diabetics-related complications [1–4]. Clinical lab indexes and fingertip blood glucose monitoring are widely used to monitor blood glucose changes over a certain period. Among the clinical glycemic parameters, HbA1c concentration can be used to measure the average blood glucose level of individuals taken over 2 to 3 months, which is the “gold standard” to measure the control of diabetes [5–7]. However, as a glycosylated product of hemoglobin, the concentration of HbA1c is affected by hemoglobin content, hemoglobin glycosylated rate, and erythrocyte clearance rate [8]. Additionally, it also does not reflect actual blood glucose changes and fluctuations, which limits its ability to measure glycemic variability and hypoglycemia [9].

In recent years, digital diabetic management has been regarded as a promising strategy, and commercial CGM devices that can record up to 10–14 days of persistent glycemic levels have been developed [10–12]. Along with the development and prevalence of continuous glucose monitoring systems (CGMS), it is predictable that indexes calculated using CGM data will have better future application prospects. CGMS provides detailed blood glucose changes [13], while indexes calculated using CGM data are under development. Some indexes evaluate a single feature of glycemic variabilities, such as standard deviation of blood glucose (SDBG), mean amplitude of glycemic excursions (MAGE), or mean of daily differences (MODD) [14–16]. In contrast, others, such as the area under the curve (AUC), low blood glucose index (LBGI) and high blood glucose index (HBGI), time in range (TIR), focus on representing a specific kind of glycemic exposure [8, 17, 18]. Although comprehensive glycemic indicators have also been proposed, none of them are as vital as the “gold standard” as to clinical decisions. There has also been some controversy regarding the integration of methods. For instance, M-value arbitrarily selects *R* as the blood glucose reference value. Simultaneously, the J-index presents the sum of elements heavily weighted towards hyperglycemia and performs poorly for hypoglycemia [4, 19].

Moreover, the choice of glycemic characters and the design of formula segmentation can have a crucial impact on these types of glycemic indicators. We found that indexes, such as the blood glucose risk index (BGRI) [20], index of glycemic control (IGC) [21], and glycemic risk assessment diabetes equation (GRADE) [22], do not independently consider glycemic variability. Additionally, BGRI and IGC do not calculate blood glucose exposure time. There are some glycemic metrics, such as continuous glucose monitoring index (COGI), Q-score, and comprehensive glucose pentagon (CGP), which cover glycemic characters more comprehensively than others [23–25]. Due to the lack of comparable evidence and common consensus, it is challenging to decide on the best-integrated method to be applied for each type of patient [26]. On the other hand, glycemic parameters, such as personal glycemic state (PGS) [27] and GRADE, are used as the piecewise function, which complicates its integration with other factors.

To develop a better method of comprehensively integrating blood glucose abnormalities, an improved algorithm of glycemic parameters has been proposed in this study. We named it the glycemic deviation index (GDI), which mainly integrates the major glycemic trend and variability. Clinical practice was considered for the design process and derivation of formulas. Methods of blood glucose scale transformation [28] and an automatically adaptive weighted adjustment method were used for GDI derivation. The duration of hypoglycemic exposure was concerned for weighting. Further, we verified the effectiveness of GDI using the simulated 24 h glycemic profiles and the CGM data of T2D participants. This glycemic index may be a potential marker that can be used to identify overall blood glucose conditions in the future.

## Methods

### Subjects

Study participants were T2D inpatients at the Department of Endocrinology at the Shandong Provincial Hospital. The institutional Ethics Committee approved the experimental protocol.

A total of 30 patients were enrolled. Patients with secondary diabetes, acute infection, stress conditions, severe organic lesions, acute diabetic complications, pregnant diabetic women, and insufficient data were excluded. The study population consisted of 13 males and 17 females between the ages of 26 to 82. The disease history of the patients ranged from less than 1 year to 20 years. Patient clinical characteristics are presented in Table 1.

**Table 1:** Clinical characteristics of type 2 diabetes patients (*n* = 30)

Clinical Characteristics	Total Results
Male(n%)	43
Age (years)	56.70 ± 14.60
History of diabetes (years)	8.43 ± 7.13
BMI (kg/m^2^)	25.00 ± 2.95
HbA1c [%(mmol/mol)]	8.99 ± 1.93
MBG (mmol/L)	10.03 ± 2.59
SDBG (mmol/L)	2.45 ± 0.98

Data are presented as mean ± SD unless stated otherwise. *BMI* Body mass index, *HbA1c* Glycated hemoglobin, *MBG* Mean blood glucose, *SDBG* Standard deviation of blood glucose

### CGMS

The CGMS (Medtronic®, iPro2) was calibrated using a minimum of four finger-prick blood glucose measurements during each 24 h period. CGMS measures the interstitial glucose level once every 5 min, and 864 recordings were obtained over 72 h. The iPro2 is composed of a CGM recorder, Sof-Sensor probe, and analysis software (CareLink iPro software Plus 1.0) in the range of 2.2 to 22.2 mmol/L. A previous study has indicated that the high accuracy of the mean absolute relative difference (MARD) of iPro2 is 9.9% for adults. In comparison, that of the Clarke error grid analysis is 99.0% (4849 of 4897) for adults [29].

### Laboratory tests

Fasting venous blood samples were drawn between 7.30 a.m. and 8.30 a.m. after 12 h or more of fasting. HbA1c levels were measured through high-performance liquid chromatography using a Tosoh HLC-723 G8. Additionally, glycated albumin (GA) levels were determined following standard methods using a Beckman Coulter AU5800 chemistry auto-analyzer. Fasting blood glucose (FBG) was calculated as the mean value of the fingertip FBG samples collected over 5–7 days and tested using an i-SENS CareSens N glucometer.

### Calculation of glycemic control parameters

Some glycemic control parameters, including mean blood glucose (MBG), the large amplitude of glycemic excursions (LAGE) [30], SDBG, MAGE, MODD, LBGI, HBGI, continuous overlapping net glycemic action (CONGA) [14], J-index and M-value, were computed using Python 3.7.0 programming. AUC_H-L_ is the mean AUC above 7.8mmol/L minus the mean AUC below 3.9mmol/L over 3 days [31]. Both AUC_H-L_ and the duration of the hypoglycemic range were calculated using iPro2 software (CareLink iPro Software Plus 1.0). The eHbA1c was calculated in Microsoft Excel using the formula: eHbA1c = 3.38 + 0.02345 × 18 × MBG (mmol/L) [32]. TIR, time below range (TBR), and time above range (TAR) were calculated based on their definitions [33].

### Design and derivation of the formulas

This study used the mean glucose index (MGI) and the standard deviation of the glucose index (SDGI) as the two main GDI components to represent CGM detected mean glucose level and its variability, respectively. MG is a fundamental element of MGI, with values ranging from 2.8 to 33.3 mmol/L based on the diagnostic criteria of hypoglycemic coma [34] and hyperosmolar coma of diabetic patients [35]. In comparison, the normal range for MG is 3.9–7.8 mmol/L, as given in basic guidelines [9]. In general, at the same level of deviation, the severity of hypoglycemia is higher than that of hyperglycemia. Therefore, we applied a method that was proposed by Kovatchev et al. to convert the blood glucose range to a clinically symmetrical form [28]. The transformation formula was used as follows, where the MG was set as “x,” while “a” and “b” were constant terms.

1-1 $$ {y}_1={\left[\ln (x)\right]}^{\mathrm{a}}-b $$

The functional form of y_1_ should meet the exponential increase of the x value at a certain symmetrical point. To ensure that y_1_ satisfies the following two criteria: 1) the value range of x is symmetrical around a certain point, 2) the target range of x is also symmetrical around this point; the following equation was constructed:

$$ {\displaystyle \begin{array}{l}\mathit{\ln}{(33.3)}^{\mathrm{a}}-b=-\mathit{\ln}{(2.8)}^{\mathrm{a}}+b\\ {}\mathit{\ln}{(7.8)}^{\mathrm{a}}-b=-\mathit{\ln}{(3.9)}^{\mathrm{a}}+b\end{array}} $$

The solution calculated using MATLAB was: a = − 0.801, b = 0.672. Inserting a and b into eq. 1–1 provided:

1-2 $$ {y}_1={\left(\ln (x)\right)}^{-0.801}-0.672 $$

**Fig. 1:**
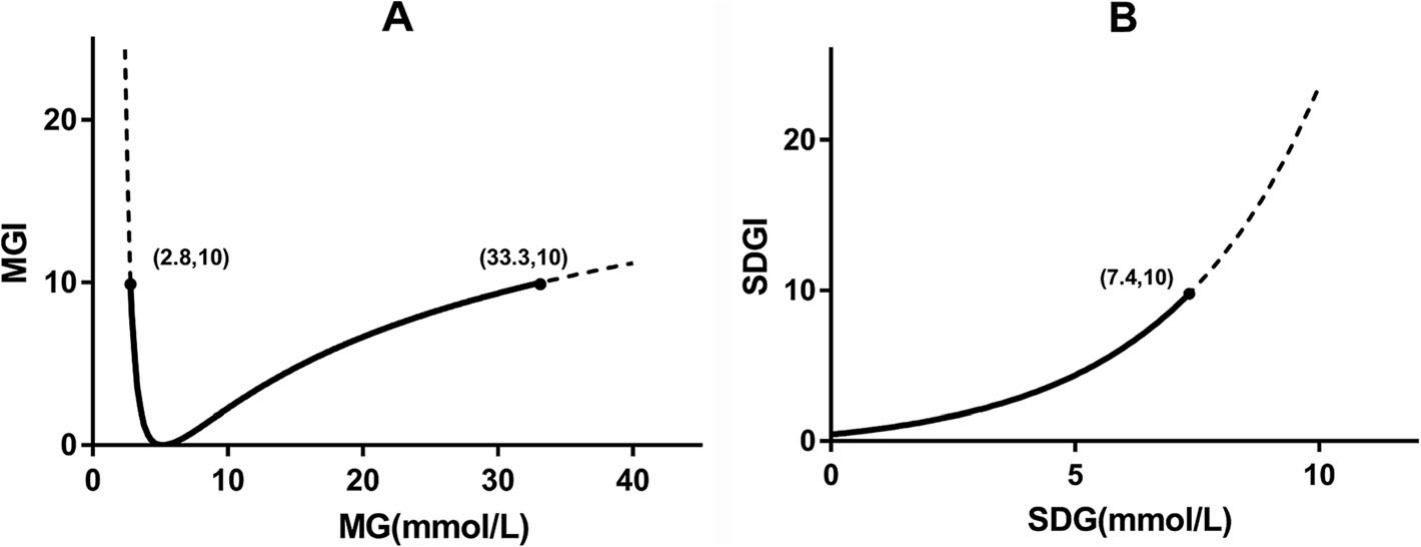
Functional images of MGI (**a**) and SDGI (**b**). The definitional domain of MGI is [2.8, 33.3], while the definitional domain of SDGI is [0, 7.4]. The value domain of both functions is [0, 10]

Furthermore, to adjust the value range to occupy the range of [0,10], and to meet the normal range at [0,1] synchronously, the following formula was introduced (Fig. 1a):

1-3 $$ {\displaystyle \begin{array}{l}{y}_2=6.337\cdot {\left(\left|{y}_1\right|+0.664\right)}^2-2.796\\ {}\mathrm{MGI}={y}_2^2\end{array}} $$

Next, to reduce the “neutralization” of hyperglycemia on hypoglycemia, MGI was calculated in two steps. MG_1_ and MG_2_ represent the average of blood glucose in the hypoglycemic and non-hypoglycemic periods separately, respectively substituted into formula 1–3 for the calculation to be performed. The weighting coefficient “c” was used to indicate duration in the hypoglycemic range (percentage of readings below 3.9 mmol/L per 72 h). The final formula of MGI was as follows:

1-4 $$ \mathrm{MG}{\mathrm{I}}^{\prime }=\mathrm{c}\cdot {\mathrm{MGI}}_1+\left(1\hbox{-} \mathrm{c}\right)\cdot {\mathrm{MGI}}_2 $$

The target range of SDG in this study was 0–1.4 mmol/L, based on previous studies, and the value range was defined as 0–7.4 mmol/L [15, 36]. The degree of deviation of glycemic variability was represented as the standard deviation of glucose index (SDGI). The SDGI value was exponentially augmented after SDG crossed over the normal threshold (Fig. 1b). SDGI was listed as follows, where “z” represented the SDG, while “e” and “f” were constant parameters:

2-1 $$ \mathrm{SDGI}={\mathrm{e}}^{\mathrm{z}}+\mathrm{f} $$

Simultaneously, the value at [0,10] should be satisfied, and the normal range was [0,1]. Therefore, the following equation was obtained:

$$ {\displaystyle \begin{array}{l}{\mathrm{e}}^{1.4}+\mathrm{f}=1\\ {}{\mathrm{e}}^{7.4}+\mathrm{f}=10\end{array}} $$

The solution was solved as e = 1.375, f = − 0.562, and these values were used in 2–1. This resulted in:

2-2 $$ \mathrm{SDGI}={1.375}^{\mathrm{z}}-0.562 $$

Then, MGI’ and SDGI were merged, and the proportions of both variables were adjusted using the adaptive weighting method. In this manner, even if one value was abnormal, and the other was normal, the abnormal value would occupy the dominant position because the ranges of MGI’ and SDGI’s were the same. The value range of the final GDI formula was [0,10], while the normal value range was [0,1], and “g” was the automatically adaptive inertia-weighted coefficient.

3-1 $$ {\displaystyle \begin{array}{l}\mathrm{g}=\frac{MG{I}^{\prime }}{MG{I}^{\prime }+ SDGI}\\ {}\\ {}\mathrm{GDI}=\mathrm{g}\cdot MG{I}^{\prime }+\left(1-\mathrm{g}\right)\cdot SDGI\end{array}} $$

Theoretically, when the patients had a normal blood glucose level, their GDI value was between 0 and 1. On the other hand, hypoglycemia, hyperglycemia, and abnormal glycemic variability resulted in GDI values higher than 1. The closer the GDI value was to 10, the worse the glycemic control would be.

### Data handling and statistical analysis

MATLAB 7.0 was used for formula derivations, and SPSS 23.0 was used for statistical analyses. The measurement data are presented as mean ± SD unless otherwise indicated. A *P* value of < 0.05 was considered to indicate statistical significance. Before one-way ANOVA was performed, non-normally distributed variables based Shapiro-Wilk testing were normalized using log-transformation (FBG) and square-root transformation (AUC_H-L_). When equal variance was not assumed (HbA1c, MODD), the Brown-Forsythe test was applied [37].

## Results

**Fig. 2:**
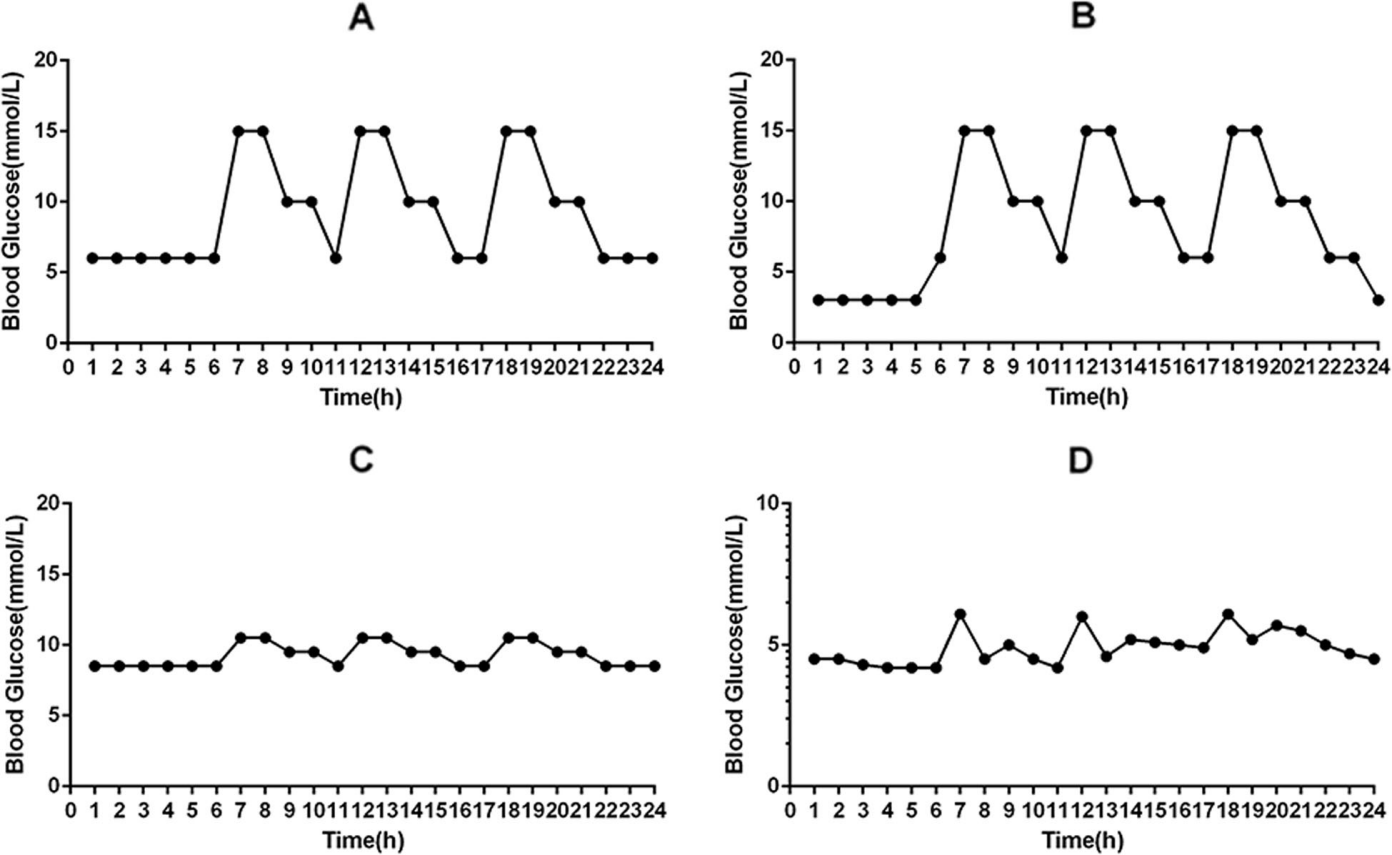
Four CGM traces were simulated for over 24 h. Hyperglycemia with high variability (**a**); hyperglycemia with high variability and the occurrence of hypoglycemia (**b**); hyperglycemia with normal variability (**c**); normal blood glucose level (**d**)

Figure 2 shows the 4 profiles of CGM over 24 h simulated under 4 typical glycemic conditions. Previous glycemic parameters (MBG, SDBG, eHbA1c, TBR, TIR, TAR) and novel parameters (MGI’, SDGI, GDI) were listed in Table 2. MBG reflects the objective average glycemic level, and SDBG reflects objective glycemic variability. eHbA1c was calculated using a method reported previously to estimate HbA1c and was listed as the clinical glycemic control reference of this study [32]. TBR, TIR, and TAR are intuitive CGM metrics that have been recommended by recent consensuses and have been thoroughly researched. The minimum and maximum glycemic level values were 3 mmol/L and 15 mmol/L, set for illustrative purposes only. Considering that postprandial hyperglycemia is prevalent in type 2 diabetes patients and increases the risk of diabetic complications [38, 39], Fig. 2a showed the fluctuating condition caused by postprandial hyperglycemia. Figure 2b demonstrated a matched high glycemic variability and average conditions with that of Fig. 2a, but with nocturnal hypoglycemia, common in diabetic patients under insulin treatment [40]. In Table 2, although both A and B showed high MBG and SDBG levels, the eHbA1c level of B was lower since the deviation of hypoglycemia was “neutralized” by hyperglycemia. However, the MGI’, SDGI, and GDI values of B were higher than that of A, using our method of overall deviation accumulation. Figure 2c simulated a condition in which the average blood glucose level was the same as in Fig. 2a, and the only difference was the glycemic variability. The results presented in Table 2 showed that C had lower SDBG, SDGI, and GDI values than A, even though the MBG, eHbA1c, TBR, TIR, and TAR values were similar. Figure 2d represented normal daily glycemic change control [41]. It showed a parallel normal glycemic variability as C. Compared with D, MBG and MGI’ were higher in C due to hyperglycemia, which also led to the rise of GDI. The GDI of D was 0.09 and was within the normal range of [0,1].

**Table 2:** Glycemic control parameters of 24 h simulated CGM data

Parameters	Model A	Model B	Model C	Model D
MBG	9.25	8.50	9.25	4.90
SDBG	3.78	4.59	0.85	0.61
eHbA1c	7.28	6.97	7.28	5.45
TBR	0%	25%	0%	0%
TIR	75%	50%	75%	100%
TAR	25%	25%	25%	0%
MGI’	1.83	3.46	1.83	0.02
SDGI	2.77	3.75	0.75	0.65
GDI	2.10	3.56	1.73	0.09

*MBG* Mean blood glucose, *SDBG* Standard deviation of blood glucose, *TBR* Time below range, *TIR* Time in range, *TAR* Time above range, *eHbA1c* Estimated HbA1c, *MGI* Mean glucose index, *SDGI* Standard deviation of glucose index, *GDI* Glycemic deviation index

Given that GDI was designed to reflect the degree of glycemic deviation comprehensively, it should contain information on each glycemic characteristic separately at the same time. To verify its characteristics, one-way ANOVA and Brown–Forsythe test were used to exam the ability of the GDI to distinguish between separate glycemic control parameters. Using the quartiles of GDI (1.2, 2.2, 2.9), we divided the 30 participants into four groups based on their GDI score: normal (GDI ≤ 1), mild (1<GDI ≤ 2), moderate (2<GDI ≤ 3), and severe (3<GDI). Table 3 shows that the lower GDI values represent better glycemic control than the higher values. The values of the clinical glycemic parameters (HbA1c, GA, FBG), average glycemic parameters (MBG, AUC_H-L_), and glycemic variability parameters (SDBG, MAGE, MODD) became significantly different as the GDI value increased. We also collected data on the average incidence of diabetic complications (diabetic retinopathy, diabetic nephropathy, diabetic neuropathies, and diabetic heart disease) for all 4 categories shown in Table 3. The results showed that the presence of diabetic complications increased as the GDI value increased.

**Table 3:** Deterioration of glycemic control positively correlated with increased GDI

Items	Normal (*n* = 4)	Mild (*n* = 9)	Moderate (*n* = 11)	Severe (*n* = 6)	*P*-Value
HbA1c (%)	6.28 ± 0.76	8.58 ± 1.66	9.55 ± 1.85	10.40 ± 0.85	*P*<0.001^#^
GA (%)	15.14 ± 2.77	23.82 ± 6.11	27.75 ± 6.21	30.73 ± 5.87	*P*<0.01^*^
log-FBG^a^ (mmol/L)	0.79 ± 0.10 (6.23 ± 1.30)	0.84 ± 0.06 (6.98 ± 0.88)	0.92 ± 0.08 (8.54 ± 1.71)	1.02 ± 0.11 (10.76 ± 2.64)	*P*<0.001^*^
MBG (mmol/L)	7.00 ± 0.27	7.88 ± 0.63	10.96 ± 1.13	13.55 ± 1.56	*P*<0.001^*^
sqrt-AUC_H-L_^b^ (mmol/L∙ day)	0.48 ± 0.32 (0.31 ± 0.21)	0.93 ± 0.20 (0.90 ± 0.39)	1.79 ± 0.23 (3.27 ± 0.79)	2.40 ± 0.29 (5.83 ± 1.53)	*P*<0.001^*^
SDBG (mmol/L)	1.06 ± 0.36	2.08 ± 0.50	2.77 ± 0.77	3.32 ± 1.01	*P*<0.001^*^
MAGE (mmol/L)	2.15 ± 0.55	3.98 ± 1.15	4.84 ± 1.49	5.27 ± 1.52	*P*<0.01^*^
MODD (mmol/L)	1.07 ± 0.47	2.07 ± 0.76	2.68 ± 0.55	3.41 ± 1.06	*P*<0.01^#^
Diabetes complications	18.75%	27.75%	33.98%	37.48%	

The results are expressed as mean ± SD. *, One-way ANOVA; ^#,^ Brown–Forsythe test. A P value of < 0.05 was considered to be statistically significant. ^a^log-transformation; ^b^square-root transformation. Original data are shown in parentheses

*HbA1c* Glycosylated hemoglobin, *GA* Glycated Albumin, *FBG* Fasting blood glucose, *MBG* Mean blood glucose, *AUC* Area under the curve, *SDBG* Standard deviation of blood glucose, *MAGE* Mean amplitude of glycemic excursions, *MODD* Mean of daily differences

## Discussion

This study aimed to develop a comprehensive index to assess the degree of deviation of blood glucose from normal levels. Therefore, a function containing the average glycemic level and glycemic variability was constructed and named as the GDI. It was composed of two main functions, MGI and SDGI, which were deduced separately. Ideally, the GDI should increase following the severity of hyperglycemia, hypoglycemia, and glycemic variability. GDI can be applied to quantitate the glycemic control of patients using CGM.

Four simulated glycemic profiles were tested to verify the efficacy of GDI, as shown in Fig. 2. The numerical results suggested that hypoglycemia, hyperglycemia, and abnormal glycemic variability could independently increase GDI values. In Table 2, MBG and SDBG, essential and widely used mathematical parameters, were computed to compare the transformed MGI’ and SDGI values [42, 43]. HbA1c is a clinical glycemic reference control, with eHbA1c as its counterpart in the simulated data. Hyperglycemia with high variability and occurrence of hypoglycemia showed a higher GDI value than the same condition without hypoglycemia, even though the MBG and eHbA1c values were at lower levels (A vs. B), indicating that hypoglycemia is an influential factor in increasing glycemic deviation in our formula. When glycemic variability or hyperglycemia was the only variable that changed in comparison with others (A vs. C, C vs. D), the GDI value remained consistent with that of the abnormal variable, indicating its ability in evaluating factors that deviate. Notably, although glycemic variability and GDI were higher (A vs. C), the TIR, TBR, and TAR remained at the same level. It indicated that the GDI could better reflect variability, while its clinical significance and the impact on diabetic complications needed to be researched further.

Additionally, we divided the thirty T2D patients into four groups based on their GDI severity to apply variance of analysis within clinical glycemic parameters, average glycemic parameters, and glycemic variability parameters. As clinical glycemic parameters, HbA1c, GA, and FBG represented glycemic control levels in separate periods [44–46]. Statistically significant differences were found among the four GDI groups, even though clinical interventions performed on patients during hospitalization were not considered. Statistical differences were also found among the four GDI groups in terms of previously established indices of the average glycemic level and variabilities, such as MBG, AUC_H-L_, SDBG, MAGE MODD. The ability to differentiate between these parameters indicated that GDI could adequately integrate corresponding glycemic information. Moreover, the increase in the average incidence of diabetic complications indicated potential correlations between GDI and diabetes complications.

Various methods have been used to evaluate overall glycemic control. However, none of them have been defined as the clinical gold standard or have been widely used in clinical practice. The TIR of 3.9–10 mmol/L, in addition to the HbA1c level, has recently been regarded as an essential metric in analyzing CGM data [9, 47]. Although TIR is not a comprehensive index, its clinical value indicates the importance of evaluating glycemic exposure time, which we have considered when designing GDI. However, specific comprehensive glycemic indicators [20, 21] have neglected this factor in their designs. In this study, the weighted coefficient “c” of MGI’ in the deduction of the GDI equation reflected glycemic exposure time (1–4). Moreover, the weighted coefficient also reflected the occurence of hypoglycemia, which proved to be effective in Fig. 2 and Table 2.

Additionally, comprehensive glycemic indicators, such as BGRI, IGC, and GRADE, tend not to add glycemic variability as an independent factor. The quantification of hyperglycemia and hypoglycemia indirectly reflected the variability, but there are some situations in which it cannot reflect. An example of this is the glycemic variability among patients with only hyperglycemia. In 1995, Wojcicki proposed a glycemic parameter to improve the M-value and named it as J-index [48]. The J-index was the first to use the idea of integrating MBG and SDBG to represent the mean level and the variability of glycemia. However, we found that without data conversion and appropriate weighting methods, hyperglycemia, hypoglycemia, and glycemic variability could interact with each other in the J-index. Thus, during the deduction of the GDI equation deducing, the standard quadratic risk function was superimposed onto the transformed blood glucose scale (1–2) [8], and value ranges were adjusted for both factors (1–3, 2–2). The adaptive weighting method was applied to ensure that the abnormal factor held the dominant position between MGI’ and SDGI (3–1).

This study has several limitations that should be noted. First, it was conducted at a single-center, which limited the number of patients who used iPro2 CGM. Second, because GDI was designed to evaluate the overall glycemic condition of individuals with CGM, it did not show transient glycemic changes. Third, there is no similar “gold standard” glycemic index, so diagnostic tests and further comparisons are required to identify GDI application limits. Finally, the relationship between the GDI and diabetic complications should be further addressed before it is applied in clinical practice.

## Conclusions

The novel glycemic parameter, GDI, is a monitoring index that integrates multiple blood glucose measures. GDI mainly quantifies abnormalities in the average glycemic level and glycemic variability based on CGM data. Therefore, the GDI may be useful for the screening and evaluation of glycemic disorders. Studies using more extensive databases are needed to investigate the application of this novel index further.

## Data Availability

The datasets generated during and analyzed during the current study are available from the corresponding author on reasonable request.

## Abbreviations

GDI: Glycemic deviation index
T2D: Type 2 diabetes
MBG: Mean blood glucose
MG: Mean glucose
CGMS: Continuous glucose monitoring system
MARD: Mean absolute relative difference
AUC: Area under the curve
BGRI: Blood glucose risk index
IGC: Index of glycemic control
PGS: Personal glycemic state
GRADE: Glycemic risk assessment diabetes equation
MAGE: Mean amplitude of glycemic excursions
SDBG: Standard deviation of blood glucose
SDG: Standard deviation of glucose
HBGI: High blood glucose index
LBGI: Low blood glucose index
LAGE: Large amplitude of glycemic excursions
MODD: Mean of daily differences
CONGA: Continuous overlapping net glycemic action
TIR: Time in range
TBR: Time below range
TAR: Time above range
GA: Glycated albumin
FBG: Fasting blood glucose
COGI: Continuous glucose monitoring index
CGP: Comprehensive glucose pentagon
